# Assessment of Sensory Processing and Executive Functions at the School: Development, Reliability, and Validity of EPYFEI-Escolar

**DOI:** 10.3389/fped.2020.00275

**Published:** 2020-05-29

**Authors:** Dulce Romero-Ayuso, Abel Toledano-González, Antonio Segura-Fragoso, José Matías Triviño-Juárez, Mª Carmen Rodríguez-Martínez

**Affiliations:** ^1^Department of Physical Therapy, Faculty of Health Sciences, University of Granada, Granada, Spain; ^2^Department of Psychology, Faculty of Health Sciences, University of Castilla-La Mancha, Talavera de la Reina, Spain; ^3^Department of Medical Sciences, Faculty of Health Sciences, University of Castilla-La Mancha, Talavera de la Reina, Spain; ^4^Primary Care Center Zaidín Sur, Andalusian Health Service, Granada, Spain; ^5^Department of Physical Therapy, Faculty of Health Sciences, University of Málaga, Málaga, Spain

**Keywords:** executive function, sensory processes, children, assessment, school

## Abstract

The aim of this study was to determine the psychometric properties of the Assessment of Sensory Processing and Executive Functions at the School (EPYFEI-Escolar), a questionnaire designed to assess the sensory processing and executive functions as underlying processes for school participation. The total sample consisted of 536 children aged between 3 and 11 years old who lived in Spain. A total of 103 teachers completed the questionnaire. An exploratory factor analysis was conducted, which showed five main factors: (1) initiation, organization, execution, and supervision of the action; (2) inhibitory control; (3) sensory processing; (4) emotional self-regulation and play; and (5) self-competence. Some of these factors were similar to those found in the EPYFEI for parents in the home context. The reliability of the analysis was high, both for the whole questionnaire and for the factors it is composed of. The results provide evidence of the potential usefulness of the EPYFEI-Escolar in school contexts for determining academic needs and difficulties of children; moreover, this tool can also be used to plan intervention programs in the school environment according to the needs of each child and school.

## Introduction

The participation of people in the different stages of life is fundamental for their development. In the case of childhood, the participation of a child in the school context is especially important (1). Several elements that can contribute to it have been pointed out, among which it is worth highlighting sensory processing (2) and executive functions (3). Disability in childhood is another risk factor that can reduce participation at the school (1).

Sensory processing refers to how the central and peripheral nervous systems organize the incoming sensory information from the sensory organs: visual, auditory, tactile, gustatory, olfactory, proprioceptive, and vestibular information (4). Three different stages can be distinguished within sensory processing: (1) detection of stimuli; (2) modulation or regulation of the intensity level of the stimuli; and (3) sensory discrimination (5, 6). Thus, it is considered that sensory processing allows registering and interpreting what happens in the environment to generate an adaptive response, integrating and processing the obtained information, and developing specific skills depending on the vital moment and on the required activity (7). From the perspective of sensory integration, sensory stimuli are considered essential for the optimal functioning of the brain, as the experiments have shown the effect of sensory deprivation on human behavior, having an even more important effect in relation to the development of specific abilities and their critical periods, for example for vision, hearing, language, etc., and to maintain optimal health status, too (8–13).

According to Dunn's model of sensory processing, four sensory profiles have been proposed for the general population as a function of the neurological threshold and the self-regulation strategies of each individual. From this perspective, the neurological threshold refers to the threshold for response to a sensory stimulus, which can be described as showing a continuous range from low to high. A person is considered to show a low sensory threshold when they notice and respond quickly to sensory stimuli. This threshold can be different for each sensory modality. Instead, it is understood that a person with a high neurological threshold requires a more intense and/or frequent sensory stimulation to notice it. Neural regulation or modulation is produced by the balance of excitation and inhibition. In Dunn's model, Thus, four types of sensory profiles have been distinguished: (1) individuals with a high neurological threshold and active self-regulation strategies, with a seeking sensory; (2) individuals with a high neurological threshold and passive self-regulation strategies, which show a bystander sensory profile; (3) individuals with a low neurological threshold and active self-regulation strategies, showing an avoider sensory profile; and (4) individuals with a low neurological threshold and passive self-regulation strategies, with a sensory sensitivity profile (14, 15). In children, difficulties in sensory processing affect their participation at all levels, with a significant impact on school activities (16). This can generate problems in social relations, since these situations require interpreting facial expression, verbal communication, and body language in order to give an appropriate behavioral response to the situation (17, 18), both in the classroom and in the schoolyard or playground. In this sense, the teaching staff could collaborate in the detection of deficiencies in executive functions and sensory processing, with the aim of understanding how these children perceive the context, in order to teach them learning strategies according to their sensory characteristics (2).

Sensory processing can be affected in multiple neurodevelopmental disorders in childhood, such as attention deficit and hyperactivity disorder (ADHD) and autism spectrum disorder (ASD), among others (19, 20). Alterations in sensory processing are present in 15% of the general population (21) and in 95% of cases in populations with neurodevelopmental disorders (22). Alterations in any of the stages of sensory processing can generate a learning dysfunction or difficulty (4). In this sense, it has been reported that there is a strong relationship between learning problems, language difficulties, sensory integration, motor problems and adaptive behavior in the classroom (23, 24). Regarding autism, several authors have found a correlation between sensory processing and repetitive behaviors (25), showing a relationship between an atypical sensory functioning in the classroom and atypical sensory responses, emotional perception and rigid thoughts, accompanied by restrictive thoughts and anxiety symptoms (26, 27). Children with autism mainly show sensory modulation problems, with different responses grouped into three patterns (28, 29): (a) sensory hyporesponsiveness (i.e., low or absent reactions to stimuli); (b) sensory hyperreactivity (high sensitivity or aversive reactions to stimuli); and (c) restricted sensory interests, repetitions, and search for behaviors (intense fascination with specific stimuli, longing for repetitive stimuli, or sensory actions based on body parts or objects). With respect to children with ADHD, sensory search patterns have been observed, which, along with the sustained attention deficit, could contribute to the emergence of difficulties in the school environment, with fluctuations in the academic performance and problems in social activities (30). It has also been reported that these children may have difficulties to identify the fingers, which has been associated with reading problems and dyscalculia (30), whereas clumsiness and the lack of motor skills, detected with graphesthesia, hinder the learning of abstract verbal concepts and calculation operations (30).

Recent studies suggest that it is fundamental for occupational therapists to expand their predominant traditional perspective, which is almost exclusively focused on understanding the difficulties in the daily functions on sensory processing/integration, in order to include executive functions (1) and reflect on the relationship between sensory deficiencies and executive functions in the performance and participation in the different activities (30). Furthermore, it has been highlighted that the traditional paradigm, which considers disorders as excluding categories, must be replaced with a different paradigm that contemplates the underlying neurobiological mechanisms, beyond a group of symptoms, to allow understanding disorders by the concurrence of phenotypes, where one symptom can be common to different disorders, such as difficulties in sensory processing (17). Thus, a strong and positive relationship has been observed between difficulties in sensory processing and deficiencies in executive functions in children with neurodevelopmental disorders (31, 32). The results of these studies show that difficulties in sensory processing and in executive functions usually come together. In this sense, it has been suggested that inhibitory control and executive attention play a crucial role in the regulation of sensory processing (33), and that tactile sensitivity can be considered as an indicator of behavioral self-regulation (34).

In the school environment, high academic performance has been associated with an optimal development of executive functions (EF), (30), especially relevant in subjects such as mathematics (35) and language (reading) (36, 37). EF are a complex set of processes that lead and monitor our actions (38). Several authors have described two types of EF: basic and advanced (39–41). Within the basic EF, three processes have been distinguished: working memory, inhibitory control, and cognitive flexibility. Regarding complex EF, planning, problem-solving, and reasoning have been included (39, 42). EF allows us to regulate our thoughts and actions in order to achieve a certain goal, in purposeful activities. For this, it is essential to keep the information active, and monitoring and updating it in our working memory in order to carry out the intended action. Inhibition let us suppress in a controlled way those distractors that can prevent us from achieving the objective of a certain task (39, 42). Likewise, EF allows a flexible behavior according to the demands of the context or activity. In daily life, in addition to basic EF, complex EF are needed, such as reasoning about the actions that will be carried out, planning and sequencing each one, and once the plan has been implemented, solving the problems that may occur in the course of time and activity (43, 44).

In summary, EF allows goal-directed behaviors, which are essential in all activities of daily living (ADL), school activities, or playing, among other human occupations. EF depend on the development and maturation of the frontal areas of the brain (45) and play a fundamental role in learning (46). In fact, one of the essential pillars of the success of classroom intervention programs is that they contemplate the development of executive functions with the aim of normalizing such behavior in the educational context, reducing the problems related to disruptive emotional, and social behaviors that could affect academic performance, such as the lack of inhibitory control, the presence of defiant conduct, and their emotional or behavioral regulation or self-control (1, 47, 48). Therefore, cognitive functions are understood as relevant skills that help children to value their performance, to be aware of their own actions and competence, and to identify and overcome possible obstacles with the aim of improving. Most children apply these skills automatically, whereas children with ADHD, for instance, require specific intervention to develop them (49). Executive functions consist of both cognitive and emotional components, and they are fundamental for the regulation of goal-targeted behavior (45, 50). Thus, they can be understood as underlying processes required for the effective performance of ADLs (51, 52), including self-directed, complex and non-routine activities in varied situations and environments (44). Therefore, further research is necessary in the field of executive functions and their influence on daily activities, highlighting the need for occupational therapists to design assessment tools for executive functions and intervention protocols, carrying out interventions based on specific evaluations that analyze the real daily performance (44). It is important to have useful tools that allow obtaining this information in an integrated way, in line with the usual childhood activities and contextualized in the school environment, since this is one of the most relevant contexts in childhood, along with play.

The study of EF in Occupational Therapy (OT) is an emerging topic in general, and specifically in children (44), as reflected the small number of instruments available to assess them (53, 54). Regarding to OT, the objective of assessment of EF is functional cognition (55). That is, the interest is to know how the different mental processes are carried out in a given context and with demands that are usually multitasking (53), rather than isolated processes, which can be better assessed with experimental laboratory tasks (54, 55). In OT, the focus is to know the impact of cognition on daily life (56), with the greater ecological validity and predictive value about functioning in the real world (57, 58).

Although there are instruments available for evaluating EF through questionnaires such as BRIEF (59), CHEXI (60), etc., these questionnaires have mainly considered cognitive processes and from OT perspective, children assessment is often focused on models of sensory processing (61, 62). However, the brain works as a whole in terms of sensory and cognitive processing, as recent studies of the human connectome show (63–65). Despite on this, and for the knowledge of the authors, there is only an instrument, developed for children aged between 3 and 11 years that assesses their participation in the different ADLs from the parents' perspective, called EPYFEI (51). This questionnaire is composed by five processes underlying the performance of ADLs: (1) attention control, working memory, and initiation of actions; (2) sensory processing; (3) emotional and behavioral self-regulation; (4) supervision, action corrections, and problem-solving; (5) inhibitory control.

All the above mentioned contributes to raising awareness about the importance of having assessment tools that allow obtaining this information and the relevance of helping the teachers to detect whether any of their students have a problem at the executive and/or sensory level, and, consequently, derive the child to the specific professional for early intervention. The aim of this study was to develop an instrument for the joint assessment of sensory processing and executive functions in children of school age, i.e., the EPYFEI-Escolar, that could be useful to teachers and occupational therapists and which would allow determining if a child had any difficulties that could affect his/her participation at the school, regardless of whether there was a clinical diagnosis.

## Methods

The methods used for the design and evaluation of the metric properties of the EPYFEI-Escolar questionnaire were based on the quality criteria for the measurement properties of health status questionnaires (66).

### Content Validity

The development of the EPYFEI-Escolar began with a literature review, followed by a meeting with three occupational therapists, three early childhood and primary education teachers and a neuropsychologist. Initially, 74 items were listed, which were based on the different theoretical dimensions of sensory processing and executive functions. Then, two rounds of consultation were conducted with three occupational therapists experts in sensory integration and four teachers (one from early childhood education, two from primary education and one from special education, all of whom worked in the public education system). In the first round, the number of items was raised from 74 to 85, the writing of some of them was modified and some autism-specific items were discarded, since the instrument to be developed was intended to be useful for the different neurodevelopmental disorders (ADHD, ASD, SLI, dyspraxia, etc…). In the second round, the questionnaire was reduced to the 80 self-administered items with which the initial form was created and with which the evaluation process was initiated. The completion of this form required between 25 and 30 min. All the consultations with the occupational therapists were conducted online and/or via phone call to verify the information provided when this was necessary. With respect to the teachers, all the consultations were carried out face-to-face. The teachers did not receive any type of compensation for participating in the study.

### Study Population

The sample was constituted by 536 children, of whom 366 were “typical” healthy children and 170 were diagnosed with some neurodevelopmental disorder (ADHD, ASD, generalized developmental syndrome, developmental delay, or other difficulties). The sample of teachers was selected from an intentional sampling of different public educational centers of the province of Toledo, which belong to the Community Government of Castilla-La Mancha (Spain), and of the provinces of Jaén, Málaga, and Granada, which belong to the Government of Andalusia (Spain). The project was initially presented to the management team of each educational center; once the interest of the study to the educational community was considered, we requested the approval of the school board of each of the participating centers. Each main classroom teacher was asked to fill in at least five questionnaires. In the case of special education teachers, they were requested to complete the questionnaires of children diagnosed with ADHD and/or ASD with known special educational needs. The main classroom teachers were required to have been in contact with each of the evaluated children for at least 3 months. This was especially important in those who went to school for the first time, since the adaptation period must be taken into account. Among the several disorders related to disruptive behaviors in school-age children, ADHD, and ASD are the most prevalent (67, 68). On the other hand, ADHD and ASD are frequently comorbid, between 50 and 80% of cases, showing an increased risk of behavioral and emotional problems (67). Furthermore, adverse consequences are especially relevant in children with ASD who do not receive support from teachers (68). Therefore, the inclusion of a clinical group and a group with neurotypical development will allow us to study the discriminant validity of the questionnaire. The field work was carried out between April 2017 and June 2019.

To analyze the repeatability and validity of the construct, 65 children from several educational centers of Andalusia were selected, whose teachers were given the EPYFEI-Escolar questionnaire between March and April 2019. Of these 65 children, the re-test was obtained in 59 cases, between 20 and 25 days after the initial administration. Furthermore, the Spanish version of the Children's Executive Functions questionnaire (CHEXI) for parents and teachers (60, 69) and the Spanish version of the Sensory Profile-2 (SP-2) for teachers, known as School Companion (14), were completed.

### Data Gathering

The participating teachers were gathered face-to-face in a first meeting, in which the purpose of the study and the questionnaire were explained and where the doubts derived from these were solved. Those who agreed to participate gave their consent and were given the questionnaire, which was required to be completed in 25–30 min. In addition to the items of the EPYFEI-Escolar questionnaire, information about the age, clinical diagnosis, school level, province, locality, and country of origin of the child was also gathered. The study was approved by the Human Research Ethics Committee of the University of Granada (code 449/CEIH/2017).

### Development of the Final Questionnaire and Internal Consistency

An initial factor analysis was conducted with the aim of identifying the important domains or concept areas, reduce the number of items if possible and determine which of them should be kept. To decide on the relevance of the factor analysis, we estimated the sample adequacy statistic of Kaiser-Meyer-Olkin (acceptable for values >0.5) and the Barlett's sphericity test. The structure was evaluated by means of an exploratory factor analysis using oblimin rotation, with maximum likelihood extraction, and applying the rule of eigenvalues >1.8 to determine the number of factors. The items were removed if they had factor loadings <0.40 with their own factor, or if they were not discriminatory for presenting similar factor loadings in several factors. The process for removing the items was to remove them one by one by performing a factor analysis repeatedly at each step. The answer options to each item (question) were based on an ordinal five-point scale (0 = never; 1 = almost never; 2 = sometimes; 3 = almost always; 4 = always), with the higher answer codes being the most favorable ones. Some items presented a very low “missing” percentage (below 0.5%), so a missing value imputation was conducted by means of a single imputation procedure. To determine the internal consistency (that is, the homogeneity of the items that measure the same attribute), Cronbach's alpha was calculated for the questionnaire and for each of the factors found in the factor analysis. A Cronbach's alpha of 0.70–0.95 was generally considered to correspond to a good internal consistency.

### Construct Validity

The validity of the construct refers to the relationship of the scores of the questionnaire with measurements of other questionnaires, in agreement with the theoretical hypotheses derived from the concepts that are being measured. To this end, the EPYFEI-Escolar questionnaire was correlated to CHEXI (60) and Sensory Profile-2 School Companion (14). CHEXI is a questionnaire aimed to evaluate executive functions in childhood, and it can be used by both teachers and parents. It consists of 24 items grouped into four factors: working memory, planning, inhibition, and regulation. This instrument has good psychometric properties, with a good internal consistency for both teachers and parents, a clear factor structure and a good predictive value on academic performance (60). On the other hand, SP-2 is a questionnaire designed to identify the characteristics of sensory processing in daily life. The Spanish version of this questionnaire can be used for the evaluation of children aged between 3 and 14 years. It consists of two models for parents (a long version and a short version, known as Short-SP-2), and one model for teachers (Sensory Profile-2 School Companion). The model for teachers consists of 44 items, distributed into five dimensions (auditory processing, visual processing, tactile processing, movement processing and behavioral response), with a reliability coefficient of 0.90 for the Spanish population, and it showed a good test-retest reliability for each profile: sensory avoiding (0.93), sensory sensitivity (0.73), sensory seeking (0.76), and low registration (0.84); school factor 1 (0.83), school factor 2 (0.67), school factor 3 (0.86), and school factor 4 (0.91) (14).

The hypothesis was that the EPYFEI-Escolar questionnaire would strongly correlate to the Sensory Profile-2 School Companion (especially to school factors 1, 2, 3, and 4, which refer to the need for support in the classroom, attention in the classroom, tolerance to the school environment and willingness to learn) and that it would show a lower correlation with the profiles of sensory seeking, sensory avoiding, sensory sensitivity, and low registration. Likewise, it was hypothesized that EPYFEI-Escolar would have a strong correlation with CHEXI. Lastly, it was established that EPYFEI-Escolar would allow discriminating between children with and without difficulties in the school context associated with their executive functions and sensory processing. To this end, Spearman's correlation tests were carried out, considering Rho >0.7 as a good value.

### Test-Retest Reliability

To determine the reliability of the questionnaire, the intraclass correlation coefficient (ICC) was used, with a 95% confidence interval, between the scores of the test and those of the re-test, in order to evaluate their temporal stability, considering ICC >0.7 as a good value.

### Floor and Ceiling Effects

In this study, floor and ceiling effects refer to the percentage of children who had the highest or lowest possible scores. The percentages of children with the highest and lowest possible scores in the total of the EPYFEI-Escolar and in each of the four dimensions were calculated. These effects were considered to be present when 15% of the participants presented minimum or maximum scores, which reduces the reliability of the instrument, since the participants with extreme scores cannot be distinguished from one another.

### Interpretability

The difference in the total score of the EPYFEI-Escolar and in the score of each of its five factors between typical (healthy) children and those with pathologies was analyzed using the Mann-Whitney's *U*-test. In addition, the ROC curve of the total score was also calculated, in order to determine the capacity of the instrument to predict whether a child is healthy or not. An additional analysis was conducted to determine the best cut-off scores. To determine the cut-off points, the coordinates of the ROC curve (sensitivity and 1-specificity) were calculated for successive scores of the total EPYFEI-Escolar score with respect to the correct classification of the child's clinical status (Healthy = neurotypical or with neurodevelopmental disorder = TEA and/or ADHD). The range of scores was between 7 and 176 points. From these data, the specificity and the Youden Index = sensitivity + specificity – 1, were calculated. The value of the EPYFEI-Escolar score corresponding to the maximum Youden index, that is, to the sensitivity and specificity, was considered as the optimal cut-off point higher.

### Statistical Analysis

Statistical analyses were performed using IBM SPSS Statistics for Windows (version 23.0, IBM Corp., Armonk, NY). Statistical significance was set at *p* < 0.05 (bilateral). The characteristics of the participants were analyzed using simple descriptive statistics.

## Results

### Sample Description

Table 1 includes descriptive data of the 536 children selected by the teachers who participated in the development of the EPYFEI-Escolar questionnaire. Of the total sample, 68.3 % (*n* = 366) were healthy children, with a majority of male children (68.3 %; *n* = 366). The average age was 7.5 ± 2.5 years (minimum 3 years, maximum 11 years), with a larger proportion of children aged between 8 and 10 years (40.7%; *n* = 218). Regarding the country of origin, 93.7% (*n* = 502) of the children were born in Spain.

**Table 1:** Sample description.

		***n^**°**^***	**%**
Group	Typical	366	68.3%
	ADHD	30	5.6%
	ASD or generalized developmental disorder	26	4.8%
	Developmental delay	11	2.1%
	Other learning difficulties	82	15.3%
	SLI	21	3.9%
Sex	Male	366	68.3%
	Female	170	31.7%
Age	≤ 4	84	15.7%
	5–7	157	29.3%
	8–10	218	40.7%
	≥11	77	14.3%
Country of origin	Spain	502	93.7%
	Other countries	34	6.3%

*SD, Standard Deviation; ADHD, Attention Deficit/Hyperactivity Disorder; ASD, Autism Spectrum Disorder; SLI, Specific Language Impairment*.

### Factor Analysis and Internal Consistency

Table 2 shows the results of the factor analysis of the five factors identified, the factor loading of each of the items, the “missing” percentage and the eigenvalues and Cronbach's alphas of the factors, as well as the explained variance after rotation. The “missing” percentage was very low in all the items (below 0.5% in all cases). A solution of five factors was reached, which were named as: (1) Initiation, organization, execution, and supervision of the action; (2) Inhibitory control; (3) Self-Regulation and play; (4) Sensory processing; and (5) Sense of competence. All the items in each factor showed a rotated factor loading over 0.4. All the factors obtained an eigenvalue above 1.8 and a Cronbach's alpha of 0.68 or higher. The total percentage of the explained variance after rotation was 62.6%. The final questionnaire derived from the factor analysis included 54 items. Given that several items of factor 1 (items 16, item 21, and items 22) had negative loadings, the data were analyzed with the inverse score for those items. The correlation between the different factors of the instrument was calculated using the Spearman's Rho test. Factor 3 (Self-regulation and play), correlated negatively with the rest of the factors (Factor 1: −0.681, *p* < 0.001; Factor 2: −0.453; *p* < 0.001; Factor 4: −0.537; *p* < 0.001; Factor 5: −0.348; *p* < 0.001), so the scale was reversed for the items in factor 3 to calculate the total scale score.

**Table 2:** Results of the factor analysis.

	**Factor loading**	**Statistic**
**FACTOR 1: INITIATION, EXECUTION, AND SUPERVISION OF THE ACTION: EXECUTIVE FUNCTIONS**		
1. Has difficulties to conduct tasks that require concentration.	0.885	Eigenvalue: 19.3
2. Requires constant efforts to conduct and finish the activities	0.823	Cronbach's alpha: 0.966
3. Has difficulties to remember necessary information when some other activity is being carried out, for instance, the mental calculation in mathematics	0.822	IC 95% (0.961–0.97)
4. Takes a long time to complete the activities. Requires more time than other children in the same age	0.816	Explained variance
5. Has difficulties to understand the necessary instructions to carry out a task when explained verbally with no visual support (blackboard)	0.809	after rotation. %: 16.2
6. Finds it hard to pay attention when performing an activity and needs to take breaks in the course of it	0.802	
7. Has difficulties to initiate and plan actions required to write or initiate an exercise	0.801	
8. Finds it hard to select essential information or materials required to perform a task or problem	0.791	
9. Has difficulties to understand the tasks regardless of how they are explained	0.789	
10. Has difficulties with the directionality and organization of space, for instance, when writing	0.761	
11. Has difficulties to follow the thread of a conversation, activity or instructions	0.730	
12. Makes mistakes due to lack of focus	0.719	
13. Has difficulties to perceive letters and words, to distinguish shapes, etc. (in paper, blackboard, etc.)	0.711	
14. Has difficulties to tell something that occurred in a way that others can easily understand it	0.709	
15. Has difficulties to coordinate eyes and hands to form letters and words, or to copy from the blackboard or book	0.699	
16. Solves the problems that emerge in the activities	−0.695	
17. Stays thoughtful, looking at nothing in particular	0.691	
18. Has difficulties to defend his/her point of view	0.688	
19. Changes activity without finishing the one that he/she was carrying out	0.688	
20. Does not realize when something changes or finds it hard to acknowledge modifications in the activity	0.619	
21. Revises and corrects the activities or tasks once they are finished	−0.615	
22. Has many ideas, is very creative	−0.594	
23. Finds it hard to go from one activity to another, regardless of whether the first one is finished, even when the teacher demands so	0.593	
**FACTOR 2: INHIBITORY CONTROL**		Eigenvalue: 3.7
24. Finds it hard to stay still	0.787	Cronbach's alpha: 0.89
25. Find it very difficult to stop carrying out activities when he/she is asked to	0.745	IC 95% (0.88–0.90)
26. Reacts emotionally in an exaggerated manner when participates in activities that involve movement	0.716	Explained variance
27. Usually hums or makes noises while conducting activities that should be done in silence	0.689	after rotation. %: 9.6
28. Tends to touch or use everything he/she sees, for instance, on the teacher's table, the classmates, etc	0.662	
29. Rocks or rocks when sitting, standing or lying	0.651	
30. Gets very excited when something special is about to happen (for instance, a school trip)	0.634	
31. Shouts or talks louder than usual regarding the context	0.620	
32. Shows difficulty avoiding laughing in situations where it is inappropriate	0.609	
33. Tries to carry out the activities that involve jumping, squeezing, pushing, or pulling, etc	0.538	
34. Shows excessive physical contact with others	0.507	
35. Conducts physical activities that involve risks, for instance, climbing, jumping from a certain height, etc	0.422	
**FACTOR 3: BEHAVIORAL – EMOTIONAL SELF-REGULATION AND PLAY**		Eigenvalue: 2.9
36. Plays adequately for his/her age in the schoolyard	0.841	Cronbach's alpha: 0.85
37. Plays with other children of the same age in playtime	0.816	IC 95% (0.83–0.87)
38. Seems to enjoy playing	0.707	Explained variance
39. Has adequate tolerance to frustration when playing	0.618	after rotation. %: 8.8
40. Recognizes the feelings and needs of others	0.584	
41. Expresses his/her feelings and needs without help	0.533	
42. Cooperates in the performance of classroom activities	0.507	
**Factor 4: SENSORY PROCESSING**		Eigenvalue: 2.2
43. Finds it hard to make eye contact with others, including the teacher, sometimes avoiding eye contact	0.599	Cronbach's alpha: 0.81
44. Is very sensitive to light	0.589	IC 95% (0.77–0.83)
45. Finds it hard to recognize objects visually	0.565	Explained variance
46. Has difficulties to recognize where the sound or voice comes from	0.559	after rotation. %: 6.1
47. Avoids activities or materials that could get his/her hands or other body parts dirty, for instance, clay, etc	0.499	
48. Seems to have little strength	0.476	
49. Is very sensitive to loud noises, showing irritation or losing track	0.469	
50. Finds it hard to keep balance	0.450	
51. Usually leans on him/herself or some object or wall to hold his/her head, body, etc	0.427	
**FACTOR 5: SENSE OF COMPETENCE**		Eigenvalue: 1.8
52. Is afraid of failure, always wanting everything to be perfect, sometimes even eliminating the will to try, due to his/her high level of rigorousness	0.703	Cronbach's alpha: 0.68
53. Is afraid of being judged, limiting his/her desire to express thoughts on the paper or verbally	0.689	IC 95% (0.62–0.72)
54. Reacts inadequately to criticism	0.499	Explained variance after rotation %: 3.1

*CI95%, confidence interval at 95% for Cronbach's alpha. Total Explained variance after rotation. %: 62.1. All items had <0.5% of missing values*.

### Discriminant Validity

Table 3 compares the Spearman's correlations between the total score of EPYFEI-Escolar and the score of each of its factors with the scores of the CHEXI and SP2 questionnaires. The results show that the total score obtained by EPYFEI-Escolar was strongly and positively correlated to each of the subscales of SP-2: sensory seeking (0.71; *p* < 0.001), sensory avoiding (0.69; *p* < 0.001), sensory sensitive (0.71, *p* < 0.001), low registration (0.72 *p* < 0.001), and behavioral response (0.79; < 0.001). Likewise, the total score of EPYFEI-Escolar showed a strong and positive correlation with the four factors of CHEXI: planning (0.76; *p* < 0.001), working memory (0.79; *p* < 0.001), regulation (0.72; *p* < 0.001), and inhibition (0.85; *p* < 0.001).

**Table 3:** Correlation between the scores of EPYFEI-Escolar, CHEXI, and SP2 (*n* = 59).

	**Total**	**Factor 1**	**Factor 2**	**Factor 3**	**Factor 4**	**Factor 5**	
Planning CHEXI	Rho	0.786	0.821	0.456	0.569	0.614	0.597
	*p*	<0.001	<0.001	0.003	<0.001	<0.001	<0.001
	*n*	41	41	41	41	41	41
Working memory CHEXI	Rho	0.807	0.816	0.511	0.588	0.613	0.583
	*p*	<0.001	<0.001	0.001	<0.001	<0.001	<0.001
	*n*	41	41	41	41	41	41
Regulation CHEXI	Rho	0.727	0.728	0.637	0.410	0.483	0.615
	*p*	<0.001	<0.001	<0.001	0.001	<0.001	<0.001
	*n*	59	59	59	59	59	59
Inhibition CHEXI	Rho	0.836	0.724	0.740	0.601	0.795	0.697
	*p*	<0.001	<0.001	<0.001	<0.001	<0.001	<0.001
	*n*	58	58	58	58	58	58
Sensory seeking profile (SP2)	Rho	0.714	0.628	0.768	0.445	0.558	0.598
	*p*	<0.001	<0.001	<0.001	<0.001	<0.001	<0.001
	*n*	59	59	59	59	59	59
Sensory avoiding profile (SP2)	Rho	0.691	0.674	0.633	0.454	0.503	0.615
	*p*	<0.001	<0.001	<0.001	<0.001	<0.001	<0.001
	*n*	59	59	59	59	59	59
Sensory sensitive profile (SP2)	Rho	0.708	0.665	0.731	0.388	0.561	0.573
	*p*	<0.001	<0.001	<0.001	0.002	<0.001	<0.001
	*n*	59	59	59	59	59	59
Low registration profile (SP2)	Rho	0.721	0.757	0.678	0.371	0.424	0.593
	*P*	<0.001	<0.001	<0.001	0.004	0.001	<0.001
	*n*	59	59	59	59	59	59
Behavioral dimension (SP2)	Rho	0.791	0.840	0.765	–.131	0.723	0.600
	*P*	<0.001	<0.001	<0.001	0.604	0.001	0.008
	*n*	18	18	18	18	18	18

### Test-Retest Reliability

Table 4 shows the test-retest differences in the total score and in the five factors of the EPYFEI-Escolar questionnaire, along with the ICC. All the differences were very small and statistically non-significant. The intraclass correlation coefficients were higher 0.9 in all the factors and in the total score.

**Table 4:** Mean scores in the test and re-test, difference and intraclass correlation coefficient.

		**Pretest**		**Retest**		**Diference**		**IC 95% DIF**				**IC 95% ICC**	
	***n***	**Mean**	**SD**	**Mean**	**SD**	**Mean**	**SD**	**Li**	**Ls**	***p***	**ICC**	**Li**	**Ls**
Total score	59	64.49	44.81	61.32	43.66	3.17	13.08	−0.20	6.54	0.068	0.98	0.96	0.99
Factor 1	59	31.15	23.23	29.32	22.23	0.98	7.89	−1.05	3.01	0.081	0.97	0.95	0.98
Factor 2	59	12.29	10.41	11.83	10.72	0.46	3.45	−0.43	1.35	0.313	0.97	0.95	0.98
Factor 3	59	18.92	7.17	8.25	5.97	0.83	4.04	−0.21	1.87	0.12	0.9	0.83	0.94
Factor 4	59	8.61	8.62	8.46	8.08	0.15	2.98	−0.61	0.92	0.695	0.97	0.94	0.98
Factor 5	59	3.36	3.55	3.46	3.48	−0.10	1.74	−0.55	0.35	0.655	0.94	0.89	0.96

*SD, standard deviation; CI 95% DIF=confidence interval at 95% of the difference; Ll, lower limit; Lu, upper limit; ICC, intraclass correlation coefficient; CI 95% ICC: confidence interval at 95% of the ICC*.

### Floor and Ceiling Effects

Table 5 shows the maximum and minimum scores of the EPYFEI-Escolar questionnaire and of its five factors, along with the percentage of individuals with maximum and minimum scores. All percentages were below 23%.

**Table 5:** Floor and ceiling effects: percentage of values in the minimum and maximum.

	***n***	**Mean**	**SD**	**Min**	**Max**	***N* in min**	***N* in máx**	**% in min**	**% in máx**
Score factor 1	536	33.46	24.22	0.00	91.00	8	1	1.49	0.19
Score factor 2	536	14.30	10.89	0.00	44.00	29	1	5.41	0.19
Score factor 3	536	8.22	6.68	0.00	28.00	1	61	0.19	11.38
Score factor 4	536	5.82	5.94	0.00	29.00	94	1	17.54	0.19
Score factor 5	536	3.11	2.88	0.00	12.00	122	5	22.76	0.93
Total Score	536	64.91	41.27	2.00	178.00	1	1	0.19	0.19

*SD, standard deviation*.

### Interpretability

**Figure 1:**
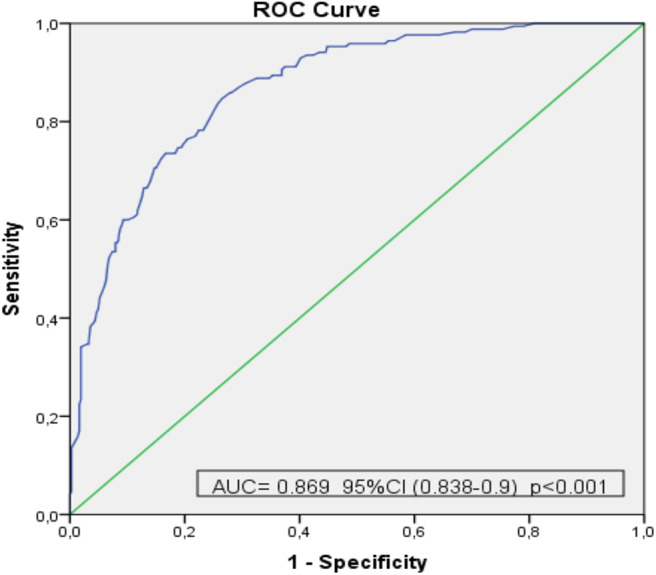
ROC curve used to determine the predictive value of the assessment instrument for sensory processing and executive functions in childhood in the diagnosis of children with a disorder.

Table 6 shows the average scores obtained by healthy children and by those with pathologies in EPYFEI-Escolar and in each of its five factors. As can be observed, there were significant differences between healthy children and children with pathologies, with higher scores among the latter and Cohen's D values considered as great differences in all the factors and in the total score of the questionnaire. Likewise, Figure 1 shows the ROC curve for the predictive level of EPYFEI-Escolar in the diagnosis of children with pathologies. The area under the curve was 0.869 (CI 95%, 0.838–0.9).

**Table 6:** Mean scores in typical children and in those with disorders.

	**Typical**			**Disorders**			**Dif**		
	***n***	**Mean**	**SD**	***n***	**Mean**	**SD**	**Mean**	**D Cohen**	***p***
Score total	366	47.78	32.99	170	101.77	32.22	53.99	1.65	<0.001
Score factor 1	366	23.42	19.87	170	55.08	17.78	31.66	1.65	<0.001
Score factor 2	366	11.29	9.30	170	20.77	11.27	9.48	0.95	<0.001
Score factor 3	366	6.27	5.87	170	12.42	6.39	6.14	1.02	<0.001
Score factor 4	366	4.25	4.66	170	9.21	6.90	4.97	0.92	<0.001
Score factor 5	366	2.56	2.53	170	4.29	3.22	1.74	0.63	<0.001

*SD, standard deviation*.

Table 7 shows the cut points of the total score of EPYFEI-Escolar as a function of the different levels of sensitivity and specificity to correctly classify healthy children and those with pathologies based on sensory processing and executive functions. The optimal cut-off score, which produced the maximum Youden's index (maximum sensitivity and specificity) was 68.5 points.

**Table 7:** Cut-off points of the assessment of sensory processing and executive functions for school questionnaire (EPYFEI-Escolar).

**Pathological if **> = ****	**Sens**	**1 - Spe**	**Spe**	**I YOUDEN**	**TP**	**FP**	**TN**	**FN**	**PPV**	**NVP**
30.5	0.976	0.593	0.407	0.384	238.3	5.7	513.0	747.0	97.6	40.7
40.5	0.953	0.481	0.519	0.472	232.5	11.5	654.1	605.9	95.3	51.9
50.5	0.912	0.393	0.607	0.518	222.5	21.5	764.3	495.7	91.2	60.7
**68.5**	**0.847**	**0.265**	**0.735**	**0.582**	**206.7**	**37.3**	**926.1**	**333.9**	**84.7**	**73.5**
80.5	0.741	0.186	0.814	0.555	180.8	63.2	1025.9	234.1	74.1	81.4
90.5	0.665	0.128	0.872	0.536	162.2	81.8	1098.2	161.8	66.5	87.2
100.5	0.565	0.085	0.915	0.480	137.8	106.2	1153.3	106.7	56.5	91.5
110.0	0.465	0.060	0.940	0.405	113.4	130.6	1184.3	75.7	46.5	94.0

*N of pathological children = 170; N of typical children = 366; Total N = 536; Sens, sensitivity; Spe, specificity; Youden I, Youden's index; TP, true positive; FP, false positive; TN, true negative; FN, false negative; PPV, positive predictive value; NPV, negative predictive value; all the data corresponding to the maximum Youden's index are in bold*.

## Discussion

In this study we explored the psychometric properties of the EPYFEI-Escolar, a new instrument to assess sensory processing and executive functions at the school. After analyzing its items, 54 of the original 80 items were retained in the final version. The results indicate that the questionnaire has good psychometric properties in terms of validity, reliability, and discriminant value for children with typical development and children with neurodevelopmental disorders. Furthermore, the design allowed the development of cut-off scores for the EPYFEI-Escolar.

The number of items in the final version of the EPYFEI-Escolar questionnaire, which was 54, was similar to that of other questionnaires for teachers about sensory processing, such as the Sensory Processing Measure (SPM) for Main Classroom Form (constituted by 62 items in the case of children in primary education and 75 items for preschool children), differing from other questionnaires, such as Sp-2 School Companion, which consists of 44 items. Regarding the SPM for Main Classroom Form, in both versions, i.e., the one for preschool children and the one for those in primary education, the items are grouped into seven theoretical dimensions: social participation, vision, hearing, touch, body awareness, balance and movement, and idea planning. The factor analysis of the SPM in the classroom for children between 6 and 11 years of age showed proprioception and the vestibular system as the principal factor for parents; a second factor comprised visual and auditory processing; another factor grouped the items of tactile processing (especially tactile hyperreaction); and two other factors included the items of praxis and social participation, with the latter being the one with the highest explanatory power in the classroom and which clearly differed from the sensory systems as a different construct (70). The SPM for preschool children showed that the factor with the highest explanatory power in the classroom was social participation, along with the factors of proprioception and praxis. Furthermore, a new factor emerged which combined items of hearing and vision, as well as one last factor about body awareness (71). The 44 items of the SP-2 for teachers, known as School Companion, are grouped into auditory processing, visual processing, tactile processing, movement, and behavioral response (14). At present, the SP2-School Companion is the only standardized instrument validated and adapted to the Spanish population that can be used to assess the sensory processing of children aged between 3 and 14 years (14). However, to the best of the authors' knowledge, there are no factor studies in the literature for the Spanish version. The dimensions that are assessed through this questionnaire emerge from the theoretical proposal of the model of sensory processing, thus the initial dimensions of the questionnaire are retained. In any case, it is worth indicating that School Companion allows determining whether or not the child requires adaptations to pay attention at school, as well as the awareness and attention of the student toward the learning environment, his/her tolerance to the conditions of the learning environment and his/her willingness to learn in such environment (14).

The results of this study suggest that EPYFEI-Escolar is an optimal instrument for detecting those students, aged between 3 and 11 years, who have difficulties in their school participation based on their sensory processing, executive functions, self –regulation and self –competence, showing a specific functioning profile, with the strengths and weaknesses of each case, thus facilitating the decision-making about educational intervention or support requirements. Numerous tests based on performance for the evaluation of executive functions have been criticized due to their lack of ecological validity (58). In this sense, EPYFEI-Escolar aims to determine the repercussion of executive functions in the school context. The items were aimed to contextualize the executive functions with the demands of the classroom and schoolyard activities, as perceived by the teachers. Thus, the factor analysis of EPYFEI-Escolar produced the cognitive dimensions of CHEXI, considered as relevant for executive functions in childhood and which are included in EPYFEI-Escolar factor 1 (69): inhibition, regulation and, jointly, working memory, and planning. There was a positive and strong correlation between the factors of both questionnaires. In addition to the dimensions recognized by other questionnaires for the evaluation of executive functions, or sensory processing, which are also contemplated in EPYFEI-Escolar, two factor emerged in this questionnaire: self-regulation and play (factor 4), which explain 8.8% of the variance and the child's sense of competence in the classroom (factor 5), which explains 3.1% of the variance.

### Reliability and Validity

The five factors of EPYFEI-Escolar demonstrated showed good internal consistency and reliability. They obtained good psychometric values for the individual's factors (attention, initiation, organization, and supervision of actions; inhibitory control; sensory processing; play and self-regulation; and self-competence) and for the total score of the questionnaire. The lowest α-scores were obtained for self-competence (0.60–0.72).

### Discriminant Validity

The EPYFEI-Escolar questionnaire and each of its factors showed good construct validity. Similarly, in a previous work with EPYFEI (51), the factor analysis confirmed two basic executive functions: “cold” executive functions, as shown by factors 1 and 2, and “hot” executive functions, related to factors 3 and 5. In addition, the interest of sensory processing (factor 4) was established.

### Interpretability

This study provides preliminary evidence of the discriminant validity of EPYFEI-Escolar. Validity was demonstrated by the fact that the scores of children with neurodevelopmental disorders significantly differed from those of children with typical development. The total score obtained for EPYFEI-Escolar makes it possible to consistently differentiate children with typical development from those with neurodevelopmental disorders and learning disabilities, with the cut-off point established at 68.5.

### Description of the EPYFEI-Escolar Questionnaire

The final scale was composed of 54 items, which were grouped into five factors: (1) attention, initiation, organization, and supervision of actions, which includes 23 items; (2) inhibitory control, with 12 items; (3) self-regulation and play, which includes 7 items; (4) sensory processing with 9 items; and (5) self-competence, with 3 items.

As in previous works, the results of this study show that executive functions represent the principal factor that contributes to the child's participation in school activities (1), obtaining two factors of executive functions (46, 72): Factor 1 (initiation, organization, execution, and supervision of the action); Factor 2 (inhibitory control); and other third factor related with self-regulation and cooperation: Factor 3 (self-regulation and play). Our results are in line with those of studies that report the higher relevance of executive functions with respect to sensory processing at explaining the participation of children in different activities, especially the activities related to school learning (1). Other authors have reported a strong correlation between executive attention and self-regulation skills (23). These results are in agreement with recent suggestions, which encourage the expansion of evaluations and treatments in pediatric OT beyond sensory processing and integration, incorporating cognitive processes, and especially executive functions (44). The fact that no different factors were found between children aged 3–5 years and those aged 6–11 years could be due to the fact that the ability to solve conflicts develops throughout the period between 2 and 5 years of age, until it reaches a level similar to that of an adult at the age of 7 years (23). Likewise, it has been suggested that there is a sequential development of the executive functions, beginning with the control of motor impulses and inhibitory control (EPYFEI-Escolar factor 2), since these are present around the age of 3 years (45, 73). Children usually achieve a good interference control at the age of 6 years, along with the development of attention, which takes place fundamentally between the age of 4 and 6 years (74), although the maturation of functions of selective and sustained attention continues. Finally, cognitive fluidity and flexibility improve progressively (75). Regarding executive functions, it has been reported that, along with planning skills (4), self-regulation abilities, such as emotional inhibition, flexibility, and regulation, are more relevant for explaining the participation of children with ASD in school activities (1). This supports the factor resolution of the EPYFEI –Escolar questionnaire for teachers, where the first two factors that explain the difficulties to participate in the classroom are basic executive functions and the third factor refers to self-regulation and play. Furthermore, executive functions predict the level of reading comprehension (76). In this sense, it has been observed that children with ADHD and executive deficiencies are as twice as likely to repeat course, compared to children with a neurotypical development (77).

With regard to factor 4 (sensory processing), visual and auditory processing have been associated with the learning of reading (78). In the case of children with ASD, auditory processing, especially auditory filtering and modulation, has been related to activities such as participation in the classroom, the use of transportation, changes between two activities, etc. (78, 79). Other sensory systems which seem to be important in school participation are the tactile system (4), specifically tactile sensitivity (78), and vestibular processing (1) or movement sensitivity (78), which has been associated with defiant behaviors. Moreover, other studies have found that, according to teachers, children with ASD show greater dysfunction in social participation and praxis (4). Likewise, recent studies have stated that praxis and social participation, along with difficulties in proprioception, seem to be more characteristic of ADHD, whereas social difficulties seem to be typical of ASD, which could be related to contextual hyperselectivity, as an inherent characteristic of ASD (80).

Our results show that factors 1, 2, 3, and 5 were positively related to CHEXI and SP2. However, there was a negative correlation between EPYFEI-Escolar factor 3, which refers to emotional and behavioral self-regulation, and the behavioral dimension of sensory profiles of SP2. This could be related to the findings of other authors, who observed that internalizing disorders, high-stress levels, anxiety, depression, shyness, and negative affection (81) are related to children with sensory processing disorders usually have learning difficulties (82). Furthermore, a strong relationship was observed between hyperactivity and the search for sensations, between inattention and the low registration profile, and between behavioral disorders and the sensory sensitive profile, although, surprisingly, the correlations were negative. That is, when the score increased in a variable it decreased in the other (21). All this indicates that specific intervention programs must be developed in order to help children with functional diversity to overcome the sensory challenges that they are facing (4, 79). Lastly, with respect to sensory processing, it has been reported that the sensory sensitive and sensory avoiding profiles are associated with lower competence in activities (83).

Regarding the self-regulation and play factor of EPYFEI-Escolar, it has been observed that the development of social skills, such as participating in cooperative plays, making eye contact with other people, keeping eye contact, recognizing and showing adequate non-verbal communication, initiating and keeping conversations, and developing long-lasting friendships, are especially sensitive aspects in children with ASD (84) and those with ADHD (72, 85). Moreover, other authors have reported that, in children with neurodevelopmental disorders (for instance, ASD, ADHD), deficiencies in executive functions, such as planning, organization, and working memory, are associated with a greater degree of isolation in the schoolyard and with difficulties at managing friendships. Children with better planning and organization skills spend more time with other children in the schoolyard (4, 86). Difficulties in social interaction are frequent in neuropsychiatric disorders. Although many processes, such as motivation and learning, contribute to establishing social behavior, the processing of external stimuli with the social context may be another important factor to consider, since all the information provided by the environment (including people and objects), is combined to compose a broad range of entities of sensory information that must be processed (17). Additionally, playing has been related to sensory processing, according to preferences for certain toys or games based on their sensory characteristics (colors, movement, sound, etc.), thus associating the sensory profile with the type of game, depending on its level of demand for activities: games with a low activity level, sedentary games, or games with a great demand for movement or physical activity (78). Moreover, children with ASD seem to require more support in social interactions (78), especially with peers, such as those which take place when playing games. Children with a low score in this factor could show difficulties at socializing with other children and participating adequately in the game with other participants, including both verbal and non-verbal communication. This type of results have been related to difficulties in sensory modulation (87). It is worth highlighting the emergence of a factor relating self-regulation and play (EPYFEI –Escolar factor 4), recognizing and expressing feelings and emotions and regulating one's behavior at school, which can be especially relevant during playtime, where the clear guideline of the teacher is usually absent and the children need to organize their own activity and behavior (4). The fact that these factors emerge in the different evaluation instruments, as it also occurs in SPM, suggests the importance of playing in the school environment (88).

These dimensions related to self-regulation are more complex from the cognitive perspective (23). In this sense, self-regulation may be understood as the ability to modulate one's behavior with the aim of achieving goals in the long term, requiring cognitive, emotional, and motivational skills (89), and that it depends on the most basic executive functions (90, 91). It is also necessary to recognize that the development of self-regulation is influenced by parenting guidelines and environmental characteristics, such as poverty, chronic stress, malnutrition, the quality of the school, groups of peers, etc. (74). The fact that emotional self-regulation emerges with playing, supports the multidimensional learning theory, which considers that children involve in more complex interactions progressively, requiring social, and emotional skills that would allow them to socialize more adequately with their teachers and classmates. This creates a positive learning environment, where they receive and give emotional support (90), while also improving other academic competences, such as the acquisition of vocabulary and mathematical skills (88, 92), thus facilitating the development of healthy habits (91).

With respect to the last factor of EPYFEI-Escolar (factor 5: sense of competence), recent studies have reported that an increase in positive self-concept in childhood is related to better executive functions (80). Self-concept is a multidimensional construct which, in the case of childhood, may be understood as the personal valuation of strengths and weaknesses, related to the child's ideal, and real performance. Positive self-concept has been related to a good academic performance. In this sense, it is understandable that the teachers included this factor in the questionnaire, due to its relevance in school participation and in the psychological development of children. Children with a low self-concept show greater internalizing disorders, such as reduced affection and feelings of despair, or externalizing disorders, such as antisocial, aggressive, and/or criminal behavior (80). In addition to emotional self-regulation, executive functions include other complex skills, such as the self-perception of competence to achieve goals and obtain a good academic performance (80), which are relevant to learning to read, decode, and understand a text, along with writing and mathematical skills, where working memory, cognitive flexibility, and inhibitory control play a fundamental role.

To the best of the authors' knowledge, there is only one questionnaire that aims to determine executive functions and sensory processing jointly, although it is limited to the scope of activities of daily living (ADL) and it can only be filled in by parents: the EPYFEI (51). This questionnaire also has five factors: (1) executive attention, working memory and initiation of actions; (2) general sensory processing; (3) emotional and behavioral self-regulation; (4) supervision, correction of actions, and problem solving; and (5) inhibitory control. The factor solution obtained for the EPYFEI-Escolar questionnaire also consisted of 5 factors, in which the factors found in the EPYFEI for parents can be included, along with two additional dimensions. The first dimension is the sense of competence, which seems to be more closely related to childhood than academic performance. The second dimension is constituted by the skills for playing and social interaction, probably due to the fact that the school is a context in which socialization takes place, along with the development of social skills required for interacting with a group of peers. This may be relevant to educational inclusion practices and the participation of children with functional diversity at the school (93). Lastly, in both questionnaires, i.e., EPYFEI and EPYFEI-Escolar, the executive functions factor is more relevant to explaining difficulties in the school context than the sensory processing factor. The factorial solution of the EPYFEI for parents is shorter, it only consists of 36 items, unlike the EPYFEI-Escolar, which has 54 items. This may be due to the greater complexity involved in participating in school activities. In the school version, the first factor of the EPYFEI-Escolar, which includes the initiation, execution, and supervision of the action, contains 23 items, while the EPYFEI for parents has only 11 items. On one hand, the development of inhibitory control seems to be more relevant in school than in ADL. At school, it constitutes the second factor (with 10 items), while at home it is the fifth factor (with 5 items) This may be because, in the classroom, children have to be in a certain posture, sitting, attentive, without moving and following the teacher's instructions, inhibiting the possible interferences of auditory or visual stimuli that are not relevant to carry out the school tasks. On the other hand, sensory processing is the fourth factor in relevance to participation in the school, unlike in ADL from EPYFEI for parents, which is the second factor in interest. In both questionnaires, emotional, and behavioral self-regulation arises in participation in the two contexts. Finally, a difference between the two questionnaires is due to the importance of participation in the school field in the development of a sense of competence. This can be explained according to developmental theories, from which it is understood that from the age of 6, the achievement of academic activities are relevant for the psychological and emotional development (94).

One of the relevant characteristics of EPYFEI-Escolar vs. other instruments that assess EF (60), is that this new tool understands that participation in the activity depends not only on the demands of the activity itself, because of the context, too. In the case of CHEXI, four factors are considered regardless of the context: working memory, planning, inhibitory control, and regulation. Instead, the BRIEF considers more factors, although always the same for parents and teachers: inhibition, self-supervision, flexibility, emotional control, initiative, working memory, planning and organization, homework supervision, and organization of materials (59).

### Implications in the Practice

One of the advantages of EPYFEI-Escolar is that it could help teachers to be more aware of the importance of the different processes that can influence the performance and participation of children in the classroom, allowing them to guide the learning strategies for each child.

Another advantage is that this tool is easy to complete, which allows conducting a relatively easy screening. Similarly, in the field of OT, the development of the EPYFEI-Escolar questionnaire proposes an advance, since, according to the best knowledge of the authors, it would be the first instrument to approach sensory processing and executive functions jointly in the school context. The creation of this tool may help occupational therapists who work in the school environment to guide teachers and parents about the best intervention strategies, in order to plan specific programs according to the needs of each child and each educational center, such as programs to improve self-regulation.

### Limitations and Future Work

The present study has some limitations. First, the socioeducational level of the parents was not obtained, which could influence in the development of executive functions and the differentiation of the results according to this variable. Second, the study did not include children over 11 years of age who were still in primary education after repeating a course at some point. Although this is a generally infrequent circumstance, it could represent a group of children with greater difficulties. However, considering that the development of EF reaches a level similar to that found in adults at about 11 years of age, we believe that including these children would not have produced significant differences in the obtained results. Third, the sample was obtained using non-probability convenience sampling. Therefore, the study must be replicated in a representative sample of healthy children and in another representative sample of children with a neurodevelopmental disorders. With respect to future research lines, it would be interesting to carry out studies in which a confirmatory factor analysis of the EPYFEI-Escolar was conducted. Another possible future line of research is to compare the results of the EPYFEI for parents and the EPYFEI-Escolar in different clinical populations. Likewise, it would be convenient to carry out studies including children with other educational needs, to determine possible profiles and provide guidelines for educational intervention, in order to improve executive functions and sensory processing. It would be interesting to analyse whether executive deficiencies would contribute to explaining the presence of sensory problems in autism or other neurodevelopmental disorders (25).

## Conclusions

The EPYFEI-Escolar questioonnaire makes a unique contribution to understanding neurodevelopmental disorders, since it considers sensory processing and executive functions simultaneously in activities carried out in the school environment.

EPYFEI-Escolar is a tool that complements other tools used by professionals who are in charge of making a more specific diagnosis and it can be a very useful instrument for teachers, since it facilitates the screening of children, allowing for the early detection of children with learning difficulties. This could help to provide a quick response to their educational needs, guiding the teacher about the strengths and weaknesses of the children regarding their executive functions and sensory processing, with the aim of optimizing the learning of the children and influencing their sense of competence, which is associated with academic success in this age range.

The psychometric results confirm the internal consistency of the instrument, as well as its construct validity and discriminant validity, according to the information provided by the participating teachers.

The factor result of EPYFEI-Escolar shows the role of multiple factors in the successful school participation, beyond academic performance, cognitive capacity, or sensory processing. EPYFEI-Escolar supports a wide perspective, and includes socioemotional competences, such as recognizing the emotions of other children and/or teachers, and responding adequately to the demands of the environment, regulating their own behavior, and emotions. All that allows developing an optimal sense of competence that could lead to the successful transition of the child to other educational stages and contexts.

## Data Availability Statement

The datasets generated for this study are available on request to the corresponding author.

## Ethics Statement

The studies involving human participants were reviewed and approved by Comité de Ética e Investigación (CEI) de la Universidad de Granada. Written informed consent to participate in this study was provided by the participants' legal guardian/next of kin.

## Author Contributions

DR-A contributed to the design and implementation of the research. AT-G and MR-M contributed to implementation of the research. AS-F analyzed the data, and all authors contributed to the writing of the manuscript.

## Conflict of Interest

The authors declare that the research was conducted in the absence of any commercial or financial relationships that could be construed as a potential conflict of interest.
